# Interactions of non-natural halogenated substrates with D-specific dehalogenase (DehD) mutants using *in silico* studies

**DOI:** 10.1080/13102818.2014.960663

**Published:** 2014-10-30

**Authors:** Ismaila Yada Sudi, Mohd Shahir Shamsir, Haryati Jamaluddin, Roswanira Abdul Wahab, Fahrul Huyop

**Affiliations:** ^a^ Department of Biotechnology and Medical Engineering, Faculty of Biosciences and Medical Engineering (FBME), Universiti Teknologi Malaysia, Johor Bahru, Johor, Malaysia; ^b^ Department of Chemistry, Faculty of Science, Universiti Teknologi Malaysia, Johor Bahru, Johor, Malaysia

**Keywords:** dehalogenases, docking, haloalkanoic acids, hydrogen-bond distance, interacting residues

## Abstract

The D-2-haloacid dehalogenase of D-specific dehalogenase (DehD) from *Rhizobium* sp. RC1 catalyses the hydrolytic dehalogenation of D-haloalkanoic acids, inverting the substrate-product configuration and thereby forming the corresponding L-hydroxyalkanoic acids. Our investigations were focused on DehD mutants: R134A and Y135A. We examined the possible interactions between these mutants with haloalkanoic acids and characterized the key catalytic residues in the wild-type dehalogenase, to design dehalogenase enzyme(s) with improved potential for dehalogenation of a wider range of substrates. Three natural substrates of wild-type DehD, specifically, monochloroacetate, monobromoacetate and D,L-2,3-dichloropropionate, and eight other non-natural haloalkanoic acids substrates of DehD, namely, L-2-chloropropionate; L-2-bromopropionate; 2,2-dichloropropionate; dichloroacetate; dibromoacetate; trichloroacetate; tribromoacetate; and 3-chloropropionate, were docked into the active site of the DehD mutants R134A and Y135A, which produced altered catalytic functions. The mutants interacted strongly with substrates that wild-type DehD does not interact with or degrade. The interaction was particularly enhanced with 3-chloropropionate, in addition to monobromoacetate, monochloroacetate and D,L-2,3-dichloropropionate. In summary, DehD variants R134A and Y135A demonstrated increased propensity for binding haloalkanoic acid and were non-stereospecific towards halogenated substrates. The improved characteristics in these mutants suggest that their functionality could be further exploited and harnessed in bioremediations and biotechnological applications.

## Introduction

**Figure 1.**
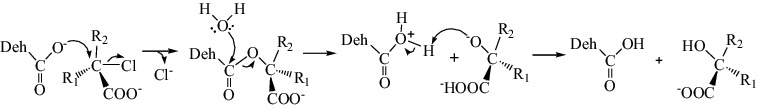
Reaction mechanism of dehalogenation catalysed by hydrolytic S_N_2-substitution reaction.

Dehalogenases have attracted a great deal of attention because of their potential applications in chemical industries and in bioremediation.[1] Bacterial D-2-haloacid dehalogenases (EC 3.8.1.9) catalyse the hydrolytic dehalogenation of D-2-haloalkanoic acids, inverting the substrate-product configuration and thereby producing corresponding L-2-hydroxyalkanoic acids.[2–7] Bacterial D-2-haloacid dehalogenases remove halogen atoms from haloalkanoic acids through different reaction mechanisms [7] and have a wide range of substrate specificity. Mechanistically, the hydrolytic substitution reaction proceeds in two steps (Figure 1). The first step is a nucleophilic attack of the α-carbon atom of 2-haloalkanoate to release a halide ion with the formation of an ester intermediate and the next step is hydrolysis of the ester intermediate to produce the corresponding 2-hydroxyalkanoates. The overall reaction involves a direct attack of the α-carbon atom of the halogenated compound by an Asp residue in the active site to form an ester intermediate and release the corresponding halide ion. The reaction is followed by an attack of the Asp residue by an activated water molecule at the γ-carbon atom in order to break the ester bond of the enzyme–product complex.[8–10] In contrast, it was proposed that group I dehalogenase directly activates a water molecule to attack the α-carbon of the 2-haloalkanoate, thereby displacing the halogen atom and not involving an ester intermediate.[11] The D-specific dehalogenases (DehDs), however, are infrequently isolated, suggesting that they are less common in nature than the L-specific dehalogenases. Hydrolytic dehalogenases have been found to occur in bacteria such as *Rhizobium* strain RC1,[12] *Pseudomonas putida* strain AJ1/23 [5] and *Agrobacterium* sp. NHG3.[13] Dehalogenases from these bacteria act at pH 8–10 and perform optimally at pH 9.5, a temperature of 50 °C and are inhibited by Cu^2+^ and most thiol reagents.[5,6]

DehD (GenBank: CAA63793.1) found in *Rhizobium* strain RC1 was cloned into *Escherichia coli*,[11] and partially purified.[14,15] The DehD hydrolyses D-2-chloropropionate, D-2-bromopropionate, D,L-2-chloropropionate, D,L-2-bromopropionate, D,L-2,3-dichloropropionate, monochloroacetate and monobromoacetate as its natural substrates.[16] Higgins et al. [13] cloned and sequenced D-haloaciddehalogenase (D-DEX) from *Agrobacterium* sp. NHG3, which was found to match closely the previously reported DehD.[3] Barth et al. [2] cloned D- and L-specific dehalogenases from *P. putida* strain AJ1/23, which shares only 15% amino-acid sequence similarity with DehD.[17] DehD is a group I α-haloacid dehalogenase and the only available representative crystal structure of a group I α-haloacid dehalogenase is that of DehI.[18] The enzyme was isolated by Senior et al. [19] from *P. putida* strain PP3 and was partially purified by Weightman et al. [20]. DehI is non-stereospecific and recognized both D- and L-substrates. To date, it has no known homologues in the structural database.

DehI has a highly basic electrostatic surface at the entrance to the active site channel relative to its molecular surface. Its functionality is to attract negatively charged haloacids to the active site entrance.[18] DehI also contains an electron-donating group located within a positively charged active site. This arrangement supports anchoring of the carboxylate residue close to the basic group, thereby promoting a nucleophilic attack on the chiral carbon.[7] The D-2-chloropropionate dehalogenation reaction involves a single inversion of the substrate configuration, as previously reported.[6] The basic residues most likely activate a water molecule for a hydrolytic attack on the α-carbon of the substrate to displace the halogen atom. Nikolic et al. [21] also highlighted the significance of the cluster of basic amino-acid residues (Arg–Lys–Arg–Arg–Arg) in the active site, which is suggested to be involved in substrate binding.

Similarly, Nardi-Dei et al. [22] proposed the first example of enzymatic dehalogenation that proceeds without the formation of a substrate-ester intermediate. The mechanism proceeds with an enzyme-activated water molecule directly attacking the α-carbon to displace a halogen atom. The reaction then proceeds with the inversion of the C_2_-configuration of the D-substrates.[23] Moreover, based on these observations, Huyop and Sudi [24] proposed a reaction mechanism for DehD in which a positively charged amino-acid residue indirectly attacks the halogen that is bound to the chiral carbon without the formation of a substrate-ester intermediate. However, this hypothesis is still subject to experimental investigation. A sequence comparison of DehI with the DehDs from *P. putida* strain PP3 and *P. putida* strain AJ1/23 shows that the amino-acid residues that line the active site are conserved, with the exception of the Ala187 residue that has been replaced by Asn in the DehDs.[18] In our previous study on DehD,[17] a total of 20 amino-acid residues were predicted to form the active site, including Val45, Met70, Thr131, Val132, Ser133, Arg134, Tyr135, Leu136, Glu138, Asp139, Ala145, Ile147, Ile148, His149, Leu150, Cys253 and Leu257. Docking of D-2-chloropropionate into the DehD active site revealed that Arg107, Arg134 and Tyr135 form hydrogen bonds with D-2-chloropropionate. The Arg107 bonded with the oxygen atom of the carboxylate group of D-2-chloropropionate (3.2 and 2.0 Å) through its 2NH1 and 1NH1 atoms, respectively. Tyr135 interacts with D-2-chloropropionate, by forming a hydrogen bond between the oxygen atoms of the carboxylate group in D-2-chloropropionate (2.7 Å) and the HH atom in Tyr135.[17] However, the interactions were weak based on the proposed hydrogen-bond distance.[25] Similarly, docking of D-2-chloropropionate into the active site of R107A, R134A and Y135A mutants reveals interactions within the limit of hydrogen bonding. Although Arg107 was not among the amino-acid residues listed in the binding site, the R107A mutant showed interactions with binding energy of 4.11 kcal/mol via its oxygen atom in the carboxylate group of D-2-chloropropionate and the NH1 atom of Arg16 (2.9 Å). The R134A mutant interacted through Tyr100 and Arg107 residues. Tyr100 interacted with the oxygen atom of the carboxylate group of D-2-chloropropionate through its hydroxyl group (3.2 Å). Meanwhile, the Arg107 in R134A mutant interacted with D-2-chloropropionate with a binding energy of 4.22 kcal/mol through two hydrogen atoms (2HH1 and 2HH2), within the proposed hydrogen-bonding distances of 2.1 Å and 1.9 Å, respectively. Lastly, the docking of D-2-chloropropionate into the Y135A mutant revealed an interaction with binding energy of 4.23 kcal/mol that occurred between D-2-chloropropionate and two residues (Arg231 and Gln221). The Arg231 residue strongly interacted with D-2-chloropropionate through hydrogen bonding at the 2HH1 and 2HH2 atoms of Arg231 at distances of 1.9 Å and 2.0 Å, respectively. The Gln221 residue of Y135A mutant interacted with the oxygen atom of the carboxylate group of D-2-chloropropionate through its 2HE2 at a distance of 2.0 Å,[17] within a hydrogen-bonding distance.[25]

Based on the above information and coupled with the fact that Arg107 was not listed among the binding-site residues, the Arg134 and Tyr135 residues in DehD were observed to interact with D-2-chloropropionate (a natural substrate of DehD), though the interaction was not strong. In lieu of this, we focused our investigation on improving the substrate-binding capabilities in Arg134 and Tyr135 by *in silico* substitutions with Ala. We investigated changes in their interactions with haloalkanoic acids and haloacetates by using docking analysis. To the best of our knowledge, the detailed reaction mechanism of DehD and the role played by each amino-acid residue remains unclear. More specifically, the involvement of the active site amino-acid residues in hydrolytic dehalogenation has yet to be demonstrated.[26] Hence, the main objective of the present study is to identify the effect of key catalytic amino-acid residue substitutions in DehD, using substrate docking into the respective mutant enzyme active site of other haloalkanoic acids (non-natural substrates upon which DehD does not act), such as L-2-chloropropionate; L-2-bromopropionate; 2,2-dichloropropionate; dichloroacetate; dibromoacetate; trichloroacetate; tribromoacetate and 3-chloropropionate. All this is aimed at increasing the catalytic efficiency of the enzyme.

## Materials and methods

### Three-dimensional structure of DehD

**Figure 2.**
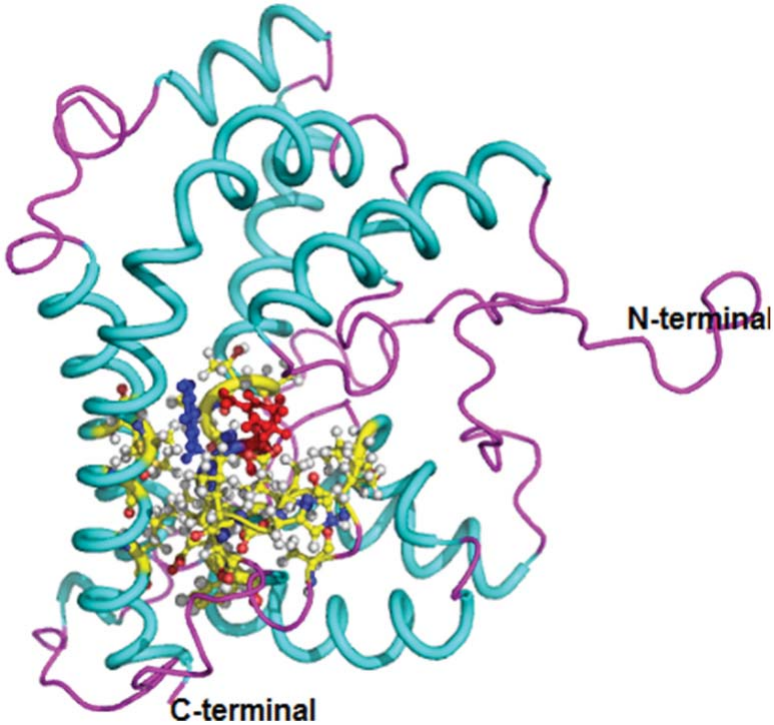
Three-dimensional structure of DehD from *Rhizobium* sp. RC1; 3D structure of DehD showing ball and stick representation of active site residues in yellow, Arg134 in red and Tyr135 in blue. (Colour version available online at: www.tandfonline.com/tbeq)

**Figure 3.**
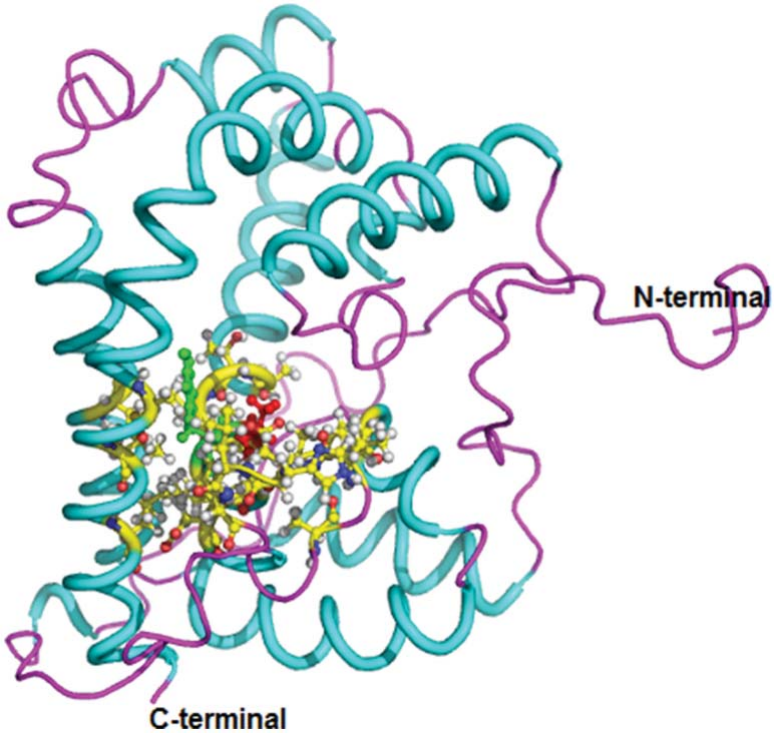
Three-dimensional structure of DehD R134A mutant from *Rhizobium* sp. RC; 3D structure of R134A mutant showing ball and stick representation of active site residues in yellow, Ala134 in red and Tyr135 in green. (Colour version available online at: www.tandfonline.com/tbeq)

**Figure 4.**
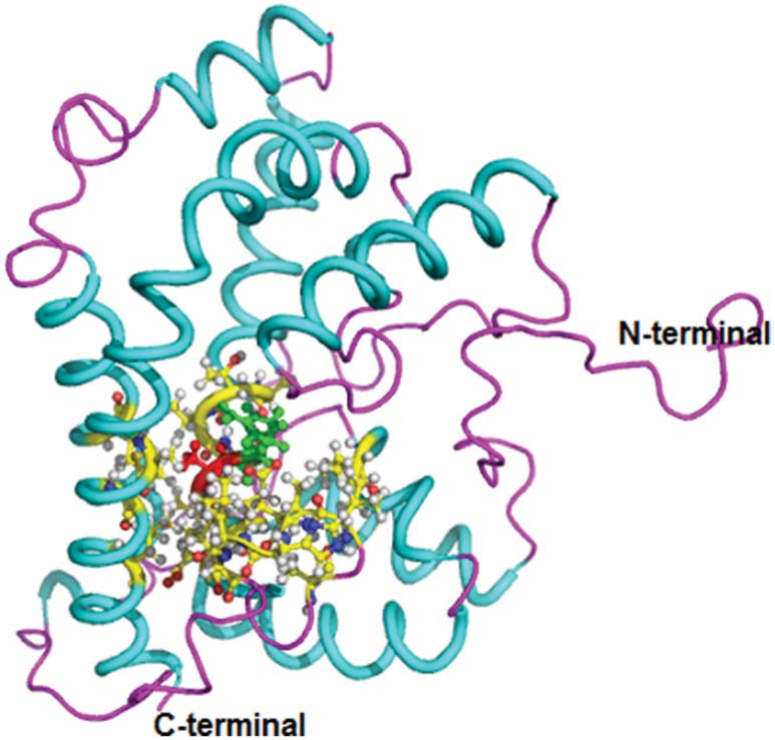
Three-dimensional structure of DehD Y135A mutant from *Rhizobium* sp. RC; 3D structure of Y135A mutant showing ball and stick representation of active site residues in yellow, Arg134 in green and Ala135 in red. (Colour version available online at: www.tandfonline.com/tbeq)

The three-dimensional (3D) structure of DehD was previously modelled [17] (Figure 2), and DehD R134A mutant (Figure 3) and DehD Y135A mutants (Figure 4) were constructed using PyMoL.[27]

### Source of halogenated propionates and acetates

**Figure 5.**
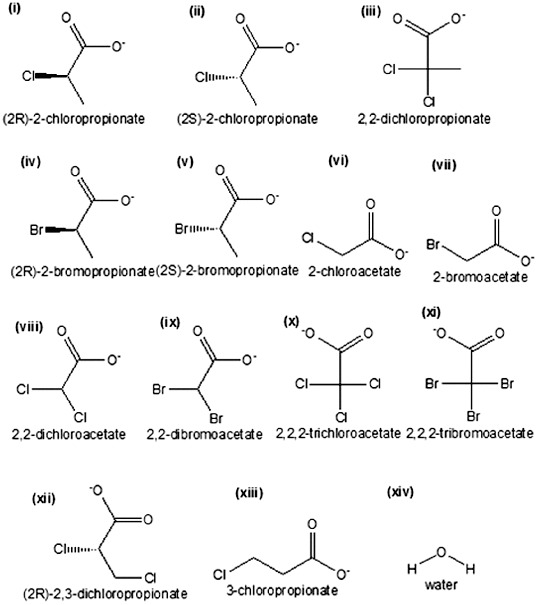
Chemical sketch of halogenated compounds and water molecule: (i) (2R)-2-chloropropionate (D-2CP); (ii) (2S)-2-chloropropionate (L-2CP); (iii) 2,2-dichloropropionate (2,2-DCP); (iv) (2R)-2-bromopropionate (D-2BP); (v) (2S)-2-bromopropionate (L-2BP); (vi) 2-chloroacetate (MCA); (vii) 2-bromoacetate (MBA); (viii) 2,2-dichloroacetate (DCA); (ix) 2,2-dibromoacetate (DBA); (x) 2,2,2-trichloroacetate (TCA); (xi) 2,2,2-tribromoacetate (TBA); (xii) (2R)-2,3-dichloropropionate (D,L-2,3-DCP); (xiii) 3-chloropropionate (3CP); (xiv) water molecule.

The substrates used in the present study include the following: L-2-chloropropionate (CID:14535399); L-2-bromopropionate (CID:6999789); 2,2-dichloropropionate, in which the hydrogen atom at the chiral carbon of 2-chloropropionate was replaced with a chlorine atom; monochloroacetate (CID:518964); monobromoacetate (CID:6226); dichloroacetate (CID:25975); dibromoacetate (CID:7269358); trichloroacetate (CID:119236); tribromoacetate (CID:6414); D,L-2,3-dichloropropionate (CID:23046803); and 3-chloropropionate (CID:6992154) (Figure 5). All substrates were downloaded from the PubChem server at http://pubchem.ncbi.nlm.nih.gov [28] and saved as PDB files using PyMOL.[27]

### Molecular docking

All molecular dockings were run using AutoDock 4.2 employing MGLTools 1.5.5, as described by Sanner [29] on an Ubuntu operating system with a 1.6 GHz quad-core processor. Atomic grid maps were computed for each atom in the ligand set. The grid centre coordinates *x* = 60.71, *y* = 69.40 and *z* = 64.42 were set so that the grid maps were centred on the DehD active site with dimensions 22 Å × 24 Å × 38 Å with a 1 Å spacing between the grid points at the centre of the protein for all atoms. Br and Cl were added to the default setting. The Lamarckian genetic algorithm (LGA) was used to search all ligand space configurations for low-energy binding orientations, thereby combining a global genetic algorithm and local minimization.[30] Non-polar hydrogen atoms were merged and torsional roots, intermolecular interactions and Gasteiger charges were assigned for all atoms of each macromolecule.[29]

The diagnostic output level for docking each ligand into the DehD active site was set at 0 for minimal output, 26 for torsional degrees of freedom for the ligand. A total of 100 runs were conducted with an initial population of 150 individuals, a maximum of 2.5 million energy evaluations, a maximum of 27,000 generations with the possibility of picking 10 worst generations, a mutation rate of 0.02 and a cross-over rate of 0.8. The resulting conformations for all molecules were re-clustered at 1 Å root-mean-square deviation (RMSD). The output file contained the predicted conformations, the orientations, the RMSD from the bound structure and the protein–ligand interaction distances for each cluster and each individual docking. The docked conformation complexes were written and analysed.[29]

## Results and discussion

### Interactions of DehD substrates with the DehD R134A mutant

**Figure 6.**
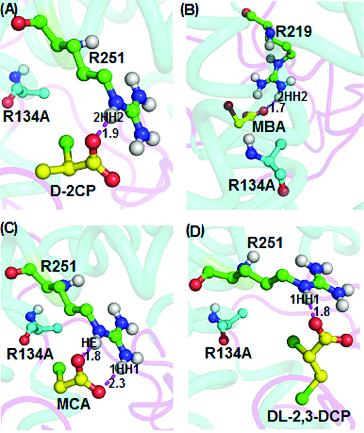
Interacting residues of DehD R134A mutant with different substrates. DehD R134A mutant hydrogen bonded to: (A) (2R)-2-chloropropionate (D-2CP); (B) 2-bromoacetate (MBA); (C) 2-chloroacetate (MCA); and (D) (2R)-2,3-dichloropropionate (D,L-2,3-DCP). The intermolecular hydrogen bonding is in magenta dashes measured in angstrom (Å). (Colour version available online at: www.tandfonline.com/tbeq)

The docking of D-2-chloropropionate, D,L-2,3-dichloropropionate, monobromoacetate and monochloroacetate into the R134A mutant active site (Figure 6) showed interactions through hydrogen bonding. Docking of D-2-chloropropionate into the active site of R134A mutant (Figure 6(A)) shows intermolecular bonding with binding energy of 4.22 kcal/mol, while Arg251 of the mutant protein interacted with D-2-chloropropionate via the 2HH2 atom through an intermolecular hydrogen-bonding distance of 1.9 Å. D-2-chloropropionate also showed a weaker interaction with the R143A mutant through the OH atom of the Tyr100 residue (3.2 Å) (not shown here). On the other hand, Arg219 in the R134A mutant gave stronger ion interaction with monobromoacetate at 1.7 Å through the oxygen atom of the carboxylate group of monobromoacetate and the 2HH2 atom of R134A at a distance of 1.7Å (Figure 6(B)). Likewise, docking of monochloroacetate into the R134A active site afforded binding energy of 4.51 kcal/mol which resulted from intermolecular hydrogen bondings of the oxygen of the carboxylate group of the ligand to the HE and 1HH1 atoms, corresponding to distances of 1.8 Å and 2.3 Å, respectively (Figure 6(C)). Additionally, docking of D,L-2,3-dichloropropionate (Figure 6(D)) also afforded an interaction with the R134A mutant with a binding energy of 4.14 kcal/mol. The 1.8 Å bond connected the carboxylate oxygen atom of D,L-2,3-dichloropropionate and the HE atom of Arg251. All these four substrates (D-2,3-chloropropionate; D,L-2,3-dichloropropionate; monobromoacetate and monochloroacetate) illustrated strong binding capabilities with the active site of the R134A mutant. These observations implied that R134A retains the substrate specificity of the DehDs and its dehalogenating potential was not compromised, as previously reported.[16,17]

**Figure 7.**
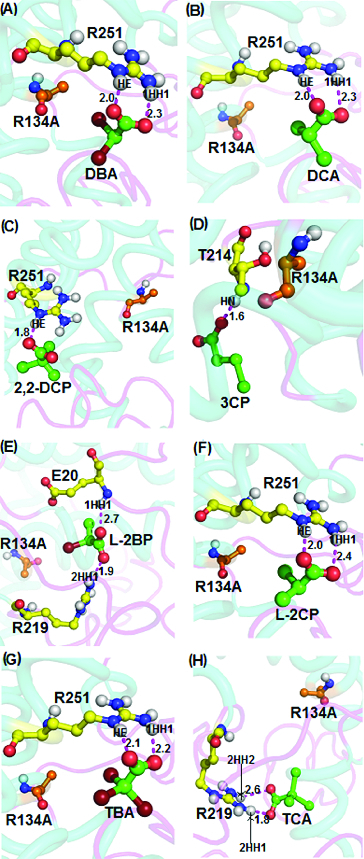
Interacting residues of DehD R134A mutant with other halogenated substrates. DehD R134A mutant bonded to: (A) 2,2-dibromoacetate (DBA); (B) 2,2-dichloroacetate (DCA); (C) 2,2-dichloropropionate (2,2-DCP); (D) 3-chloropropionate (3CP); (E) (2R)-2-bromopropionate (L-2BP); (F) (2S)-2-chloropropionate (L-2CP); (G) 2,2,2-tribromoacetate (TBA); and (H) 2,2,2-trichloroacetate (TCA). The intermolecular hydrogen bonding is in magenta dashes measured in angstrom (Å). (Colour version available online at: www.tandfonline.com/tbeq)

Next, non-natural wild-type DehD substrates, namely, halogenated propionates and acetate, were docked into the R134A mutant active site (Figure 7). In all cases, docking of dibromoacetate, dichloroacetate, L-2-chloropropionate and tribromoacetate into the active site revealed binding energies of 4.15 kcal/mol, 4.41 kcal/mol, 4.18 kcal/mol, 3.77 kcal/mol, respectively, and with strong intermolecular hydrogen-bond distances between 2.0 and 2.4 Å. The bondings occurred between the HE atom of the R134A mutant with the carboxylate group of the ligand, while the 1HH1 atom was predicted to interact with the carbonyl group of Arg251 (Figure 7(G)) and with 2HH2 of Arg219 in intermolecular bonding involving trichloroacetate and R134A mutant (Figure 7(H)). On the other hand, the substrates 2,2-dichloropropionate, 3-chloropropionate and L-2-bromopropionate formed intermolecular hydrogen bonding with the R134A with binding energies of 4.41 kcal/mol, 4.33 kcal/mol and 4.21 kcal/mol, respectively. The 2,2-dichloropropionate, 3-chloropropionate and L-2-bromopropionate formed strong intermolecular hydrogen bonds with the R134A mutant through oxygen atoms of the carboxylate groups and the HE atom of Arg251 (Figure 7(C)), the HN atom of Thr214 (Figure 7(D)) and the 2HH1 atom of Arg219 (Figure 7(E)), with a length of 1.8 Å, 1.6 Å and 1.9 Å, respectively. Additionally, Glu20 strongly bonded with L-2-bromopropionate at a distance of 2.7 Å (Figure 7(E)).

Docking of trichloroacetate into the active site of the R134A mutant gave a binding energy of 4.08 kcal/mol and both the 2HH1 and 2HH2 atoms of R134A via the Arg219 residue interacted with the oxygen atoms of carboxylate and the carbonyl groups of trichloroacetate at distances of 1.8 Å and 2.6 Å, respectively (Figure 7(H)). The R134A mutant exhibited strong interactions with the ligands as the recorded intermolecular hydrogen bonds were within acceptable (2.6 Å) bonding distance. These observations indicate that the R134A mutant shows potential to degrade L-substrates and 3-chloropropionate, unlike the wild-type DehD.[11,16] Remarkably, the strong interaction of R134A with 3-chloropropionate is a consequence of a significantly shorter hydrogen bond of just 1.6 Å (Figure 7(D)). It can be considered that the mutation of R134 to Ala rendered the enzyme more competent in degrading 3-chloropropionate.

### Interactions of DehD substrates with the DehD Y135A mutant

**Figure 8.**
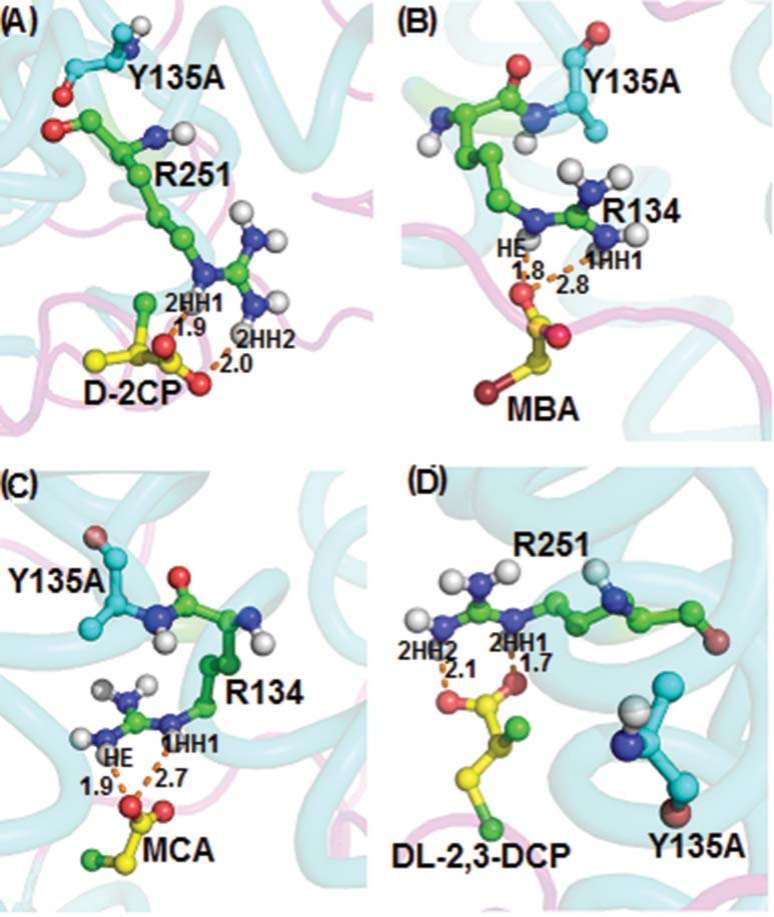
Interacting residues of DehD Y135A mutant with different substrates. DehD Y135A mutant bonded to: (A) (2R)-2-chloropropionate (D-2CP); (B) 2-bromoacetate (MBA); (C) 2-chloroacetate (MCA); and (D) (2R)-2,3-dichloropropionate (D,L-2,3-DCP). The intermolecular hydrogen bonding is in orange dashes measured in angstrom (Å). (Colour version available online at: www.tandfonline.com/tbeq)

Docking of monobromoacetate, monochloroacetate and D,L-2,3-dichloropropionate into the active site of the Y135A mutant (Figure 8) showed several hydrogen-bonding interactions. Docking of D-2-chloropropionate into the active site of the R135A mutant gave a binding energy of 4.23 kcal/mol (Figure 8(A)) arising from intermolecular hydrogen bonds between 2HH1 (1.9 Å) and 2HH2 (2.0 Å) of Arg251 with D-2-chloropropionate. Arg134 in the Y135A mutant interacted through its HE and 1HH1 atoms with the oxygen atom of carboxylate of monobromoacetate at distances of 1.8 Å and 2.8 Å, respectively (Figure 8(B)). Monochloroacetate, when docked into the active site of the Y135A mutant, resulted in a binding energy of 4.64 kcal/mol from interactions via its carboxylate group oxygen atom with the HE and 1HH1 atoms of Arg134 which corresponded to 2.7 Å and 1.9 Å, respectively (Figure 8(C)). Interaction of D,L-2,3-dichloropropionate with the Y135A mutant afforded binding energy of 4.05 kcal/mol via its carboxylate oxygen atom with 2HH1 and 2HH2 of Arg251 at 1.7 and 2.1 Å, respectively (Figure 8(D)).

Meanwhile, docking of natural DehD substrates (D-2-chloropropionate, D,L-2,3-dichloropropionate, monobromoacetate and monochloroacetate) into the active site of the DehD Y135A mutant revealed that their binding occurs through the Arg134 residue. Although the binding energy of monobromoacetate was 4.49 kcal/mol, 3.31% lower than that of monochloroacetate, it was observed that both substrates formed strong (1.8 and 1.9 Å) and moderate (2.8 and 2.7 Å) hydrogen bonds with Arg134 in the Y135A mutant, respectively. Likewise, D,L-2,3-dichloropropionate also exhibited strong interactions (1.7 and 2.1 Å) with Arg25. Previously, we reported that the wild-type DehD interacts with the carboxylate oxygen atoms of monobromoacetate and monochloroacetate through nitrogen (NH1 and NH2) atoms of Arg107 in the Y135A mutant, resulting in significantly longer hydrogen-bonding distances of 2.9 and 2.7 Å, respectively.[17] Mutation resulted in the R135A mutant to form stronger bonds with D,L-2,3-dichloropropionate than previously described in wild-type DehD.[17] The findings indicate that halogenated substrates bonded favourably to the mutant protein active site and the Y135A mutant could be a better dehalogenating enzyme, while retaining its catalytic activity.

**Figure 9.**
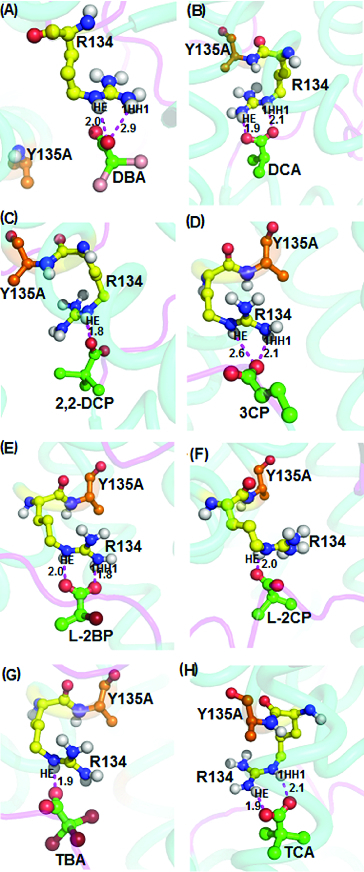
Interacting residues of DehD Y135A mutant with other halogenated substrates. DehD Y135A mutant bonded to: (A) 2,2-dibromoacetate (DBA); (B) 2,2-dichloroacetate (DCA); (C) 2,2-dichloropropionate (2,2-DCP); (D) 3-chloropropionate (3CP); (E) (2R)-2-bromopropionate (L-2BP); (F) (2S)-2-chloropropionate (L-2CP); (G) 2,2,2-tribromoacetate (TBA); and (H) 2,2,2-trichloroacetate (TCA). The intermolecular hydrogen bonding is in magenta dashes measured in angstrom (Å). (Colour version available online at: www.tandfonline.com/tbeq)

Next, docking of non-natural halogenated propionates and acetates of DehD substrates into the active site of Y135A mutant (Figure 9) showed that the oxygen atom of the carboxylate of dibromoacetate interacts with a binding energy of 4.11 kcal/mol via the HE and 1HH1 atoms of Arg134 at 2.5 and 2.9 Å, respectively, whereas interaction of carboxylate groups with the substrates 2,2-dichloropropionate, L-2-chloropropionate and tribromoacetate, with the same atoms, predicted significantly shorter distances of 1.8, 2.0 and 1.9 Å, respectively (Figure 9(C), 9(F) and 9(G)). Similarly, the oxygen atom of the carboxylate of 3-chloropropionate bonded with the HE and 1HH1 of Arg134 at distances of 2.6 Å and 2.1 Å, respectively (Figure 9(D)).

It was observed that dichloroacetate, L-2-bromopropionate and trichloroacetate form hydrogen bonds (1.8 to 2.1 Å) through the HE and 1HH1 atoms with the oxygen atom of the carboxylate group, as well as the oxygen atom of the carbonyl group, respectively (Figure 9(B), 9(E) and 9(H)). Our analyses indicated that Arg134 was the common residue that interacted with all substrates, with the exception of D-2-chloropropionate and D,L-2,3-dichloropropionate, which interacted with Arg251. The hydrogen-bond distances were also within the favourable distance (<2.6 Å),[25,31] suggesting that the Y135A mutant can degrade both L- and D-substrates. The involvement of Arg134 in the binding of halogenated substrates in DehD mutants implies that this residue activates a water molecule to attack the α-carbon of the substrates, in accordance with the proposed mechanism of enzymatic hydrolytic dehalogenation of haloalkanoic acids without formation of an ester-intermediate.[4,6,18,22]

However, the degradation of 3-chloropropionate or β-chloro-substituted alkanoates is not well documented. So far, there have been reports describing non-stereospecific (DehE) and stereospecific (DehD and DehL) dehalogenases unable to degrade 3-chloropropionate and some limited reports on bacterial degradation of β-substituted haloalkanoic acids. Hirsch and Alexander [32] observed that the β-substituted propionates are degraded more slowly than the α-substituted compounds. However, only Rhodospirillum rubrum, *R. photometricum* and *Rhodopseudomonas palustris* grow on 3-chloropropionate.[33] In addition, 3-chloropropionate is also degraded by several bacteria, namely *Pseudomonas sp*. B6P [34,35] from paddy agricultural soil and *Rhodoccoccus* sp. HJ1 [36] with similar activity towards 3-chlorobutyric and 2,2,3-trichlorobutyric acids. Recent *in silico* investigation by rational design of DehD confirmed the involvement of Arg16 and Arg134 in the specific binding to (2R)-2-chloropropionate,[37] while this *in silico* analysis showed that a mutation at R134 and Y135 could render the enzyme able to degrade other non-natural substrates. However, it was determined experimentally that DehD does not degrade 3-chloropropionate (3CP) [9,13], nor does it show any interaction during docking studies.[17]

In this context, our study showed that the DehD mutants R134A and Y135A afforded strong hydrogen-bonding interactions with 3-chloropropionate, implying that these mutant dehalogenases could be considered as viable biotools for reducing the toxic effect of 3-chloropropionate on human health and the environment. The binding of 3-chloropropionate to the DehD R134A and Y135A mutants at short bonding distances suggests that these enzymes have a great potential for future development, and represents, to the best of our knowledge, the first reported case for *Rhizobium* sp. RC1 which could have unlimited applications in bioremediation and biotechnology.

## Conclusions

In summary, the present study demonstrated the different hydrogen-bonding capabilities of DehD R134A and Y135A mutants with haloalkanoic acids during interaction with stereo-haloalkanoic acids. Remarkably, the mutants exhibited a strong interaction with 3-chloropropionate at Arg134, in the light of very few reports on bacteria able to degrade 3-chloropropionate. We believe that DehD mutants have great potential to degrade both L- and D-substrates. That is to say, the DehD mutants are non-stereospecific towards halogenated substrates, and thus become economically attractive to serve as enzymes with diverse applications in in the field of bioremediation and biotechnology. Further rational design experiments using site-directed mutagenesis must be performed to ascertain the great potential of the D-2-haloacid dehalogenase from *Rhizobium* sp. RC1.
